# Perceptions and satisfaction of caregivers regarding rehabilitation services from selected rehabilitation centres in the Western Cape

**DOI:** 10.4102/ajod.v7i0.415

**Published:** 2018-10-25

**Authors:** Nondwe B. Mlenzana, Arne H. Eide, Jose M. Frantz

**Affiliations:** 1 Faculty of Community and Health Sciences, University of the Western Cape, South Africa; 2 Faculty of Medicine and Health Science, University of Stellenbosch, South Africa; 3 SINTEF, Oslo, Norway

## Abstract

**Background:**

Understanding caregivers’ views on rehabilitation services is important as it may assist in informing healthcare services and patient management.

**Objectives:**

The aim of this study was to explore caregivers’ perceptions and satisfaction regarding rehabilitation services in the Western Cape, South Africa, and to inform clinical practice and policy in this emerging field.

**Method:**

This study used a descriptive, qualitative design using in-depth interviews with conveniently selected participants. Interviews were conducted with 13 caregivers of patients with: amputations (3), cerebrovascular accidents (5) and neuromuscular disorders (5). Thematic content analysis was conducted with the transcripts.

**Results:**

Four key themes emerged, which were (1) financial difficulties, (2) caregiver and therapist relationships, (3) facility management and (4) caregiver experience with service delivery.

**Conclusion:**

Based on the participants’ feedback, the rehabilitation services seem to be meeting the basic rehabilitation needs of the patients; however, the needs of the caregivers require attention.

## Introduction

Access to rehabilitation is, according to the United Nations Convention on the Rights of Disabled People (CRDP) (UN 2006), a right for persons with functional problems (Article 26). However, according to the World Report on Disability (WHO 2011), such services lack both in quantity and quality in low- and middle-income countries (LMICs). Research on rehabilitation in LMICs may be one of the entry points for improved access and quality of services. The role of rehabilitation cannot be underestimated in the reintegration of the patient into his or her community. Caregivers often play a central role in determining the success of the rehabilitation process (Alexandrou 2014). However, caregivers’ perspectives on rehabilitation are often side-lined, both in practice and in research (Tin, Mehta & Rowlands 2006). To improve rehabilitation services in LMICs, it is necessary to hear the voices of caregivers in addition to the users themselves and professional rehabilitation workers. This study sets out to explore caregivers’ perspectives on rehabilitation within the context of rehabilitation services in the Western Cape, South Africa.

The Integrated National Disability Strategy in South Africa defines rehabilitation as ‘ways of helping people with disabilities to become fully participating members of society, with access to all the benefits and opportunities of that society’ (Office of the Deputy President 1997, Chapter 3), including not only the patient and the health professionals but also the caregiver as a key contributor to rehabilitation (Witness 2010). For effective rehabilitation, excellent communication is required between the health professionals regarding the patient and, in many instances, the caregiver becomes the channel of communication. In addition, rehabilitation professionals rely on caregivers to become extensions of the intervention programmes and to ensure that exercise takes place at home. As the caregiver becomes co-manager of the patient’s care plan, it is essential that the caregiver understands the patient’s needs and challenges. Feedback from patients and other key stakeholders, such as caregivers, is required if deficiencies are to be identified and improved. In addition, it is important that the impact of the caregiving process is understood to provide rehabilitation professionals with insight to facilitate this process.

The burden experienced by caregivers is usually considered to be both task-oriented as well as emotionally challenging (Edwards 2008). The actual tasks performed by the caregiver are dependent on the physical dependency of the patient and the emotional challenge is influenced by the mental and emotional health of the patient (Edwards 2008). Therefore, Edwards (2008) emphasises that the needs of the caregivers must be understood and supported. Ability to cope with both the practical tasks and the emotional challenges is paramount to achieving a healthier caregiver and thus, ultimately, a healthier patient (Edwards 2008).

In addition to understanding the needs of the key role players in rehabilitation, it is important to obtain their assessment of and to what extent they are satisfied with the services provided. While customer satisfaction in the marketing industry is linked to increased sales and the generation of profits (Woodside et al. 1998), in healthcare it ensures the provision of quality outcomes, which is the goal of every health facility (Steinwachs & Hughes 2008). It is highlighted in studies carried out in different countries that healthcare providers find it challenging to satisfy the needs of the patients. Often, providers do not meet specific and expected outcomes of service delivery as there are shared barriers to these outcomes (Crisp 2000; Van Til et al. 2010; Williams & Bowie 1993). Because of the multidimensional nature of satisfaction, researchers have, over time, continued to develop models to explain the factors that influence satisfaction (Conway & Willcocks 1997; Fiebelkorn 1985; Woodside et al. 1998).

Based on literature (Crisp 2000; Mlenzana et al. 2013), it is envisaged that within the health setting, patients enter a service setting with needs, wants and expectations. The extent to which the provider fulfils them defines the degree to which the patient will be satisfied. Research regarding the needs of the caregiver and the barriers to and the facilitators of the caring process is scarce. According to Kruzich et al. (2003), caregiver involvement in the rehabilitation process requires attention. However, research evidence suggests that support for caregivers is often not forthcoming. Studies have been conducted in South Africa focusing on patient and service provider experiences with rehabilitation services (Kumurenzi et al. 2015), as well as on caregivers of children with disabilities (Saloojee 2011). Thus, the current study builds on the available knowledge regarding perceptions of rehabilitation services by exploring the perceptions and satisfaction of caregivers of adult disabled patients regarding rehabilitation services in the Western Cape, South Africa. The research question is: What are the perceptions and levels of satisfaction of caregivers regarding rehabilitation within the context of selected rehabilitation centres in the Western Cape, South Africa?

## Methods

### Design

The study used an exploratory, descriptive, qualitative design, using in-depth interviews to explore the perceptions and satisfaction of caregivers regarding rehabilitation services from selected rehabilitation centres in the Western Cape.

### Sample

The target population and inclusion criteria for this study involved all caregivers who volunteered to accompany people with disabilities to selected rehabilitation centres in the Western Cape, South Africa. The rehabilitation centres were selected based on the fact that they were primary healthcare centres as well as the populations that they service. People with disabilities are consulted through a referral system or through self-referral to the rehabilitation centres and they do not pay for services as healthcare services should be accessible for all. The sample consisted of caregivers of 13 individuals, conveniently selected from those patients with physical disabilities who had received rehabilitation services at the identified centres. The persons receiving rehabilitation at the centres include those with conditions such as stroke, amputations, head injuries, spinal cord injuries and neuromuscular disorders leading to disability.

### Instrumentation

An interview guide that consisted of open-ended, non-directive questions was used to explore the caregivers’ perceptions of and satisfaction with the rehabilitation services that their family member was receiving. An initial open-ended question was used to explore the caregivers’ perceptions of the rehabilitation service. Probes were used to obtain in-depth descriptions of their perceptions and satisfaction with the services. To ensure trustworthiness (Lincoln & Guba 1985) in this study, the interview guide was developed after the researcher conducted a systematic review, which focused on barriers and facilitators to rehabilitation services (Mlenzana et al. 2013). Themes that emerged from the systematic review were: financial difficulties, caregiver and therapist relationship, facility management and caregiver experience with service delivery, and were used as predetermined themes of this study. The interview guide was developed and reviewed by individuals considered experts in the area of disability and rehabilitation. Participants who were recruited were fluent in English and Afrikaans.

### Procedure

Twenty-six suitable participants were telephoned and, following an explanation of the purpose of the study, were asked to participate. However, of these participants, only 13 were available for interviews and some indicated a willingness to participate but did not arrive on the particular day. An appointment to conduct the interviews was made with those who agreed telephonically to participate in the study. The researcher followed up on the participants who did not arrive, and they stated that they had other commitments on the day of the interview. Informed written consent was obtained from all participants before each interview was conducted. Participants were assured of anonymity and their right to withdraw from the study. In addition, permission to audio-record the interview was also obtained from the participants. Interviews were conducted during June 2011, using in-depth interviews, and were carried out in the caregivers’ homes or at the rehabilitation centres at a time that was suitable for them. The audio-recordings were transcribed verbatim. The participants were given an option as to the language in which the interviews could be conducted. Eight of the interviews were conducted in Afrikaans. The research assistant who was with the researcher was fluent in both English and Afrikaans. Interviews were conducted in both languages to accommodate the group and the interview guide was prepared in both English and Afrikaans.

### Data analysis

All taped interviews were transcribed verbatim. The transcripts were then compared to the voice recordings to verify accuracy. The Afrikaans transcripts were translated into English after the recordings were verified. Transcriptions were translated from English and back to the interviewees’ language to ensure validity. Data were analysed using a coding process to sort the information according to categories within the predetermined themes. Data analysis was performed using the following predetermined themes: financial difficulties, caregiver and therapist relationship, facility management and caregiver experience with service delivery. Within the predetermined themes, categories were identified by the first author and consensus was reached through discussion with the second author. All categories were supported with quotes from the interviews.

### Ethical considerations

Ethical clearance to conduct the study was obtained from the Senate Research Ethics Committee of the University of the Western Cape, reference number: 10/1/3.

## Results and discussion

### Characteristics of the participants

The study sample consisted of 13 participants with a mean age of 47 years for the caregivers and 59 years for the patients. The majority of the caregivers were female and in most instances the spouse of the patient (Table 1).

**TABLE 1a:** Caregiver demographics.

Age	Gender	Relation to patient
70	Female	Wife
58	Male	Husband
56	Male	Husband
34	Female	Niece
27	Female	Aunt
45	Female	Daughter
29	Male	Friend
56	Male	Brother-in-law
27	Female	Daughter
63	Male	Husband
46	Female	Neighbour
34	Female	Neighbour
60	Female	Daughter

**TABLE 1b:** Patient demographics.

Age	Gender	Condition
77	Male	Amputation
58	Female	Amputation
54	Female	Amputation
68	Male	Cerebrovascular accident
12	Female	Neuromuscular disability
67	Female	Cerebrovascular accident
28	Male	Neuromuscular disability
65	Female	Neuromuscular disability
59	Female	Cerebrovascular accident
58	Female	Cerebrovascular accident
68	Female	Neuromuscular disability
64	Male	Cerebrovascular accident
84	Female	Orthopaedics

### Main findings

The four main emerging themes and categories are presented in Table 2. Quotes to support the themes will be presented below.

**TABLE 2:** Emerging themes and categories.

Theme	Category
Financial difficulties	Transportation cost to the rehabilitation service (not having money).
Caregiver and therapist relationship	Caregiver integration in the rehabilitation process. Exchange of information between the therapist and the caregiver. Trust in the relationship between the caregiver and therapist.
Facility management	Good access to rehabilitation services. Challenge in accessing files prior to therapy. Insufficient frequency of appointments, that is, long waiting periods between follow-up sessions.
Caregiver experience with service delivery	Physical health: seldom physically capable to assist the patient. Emotional health: neglect own emotional needs.

### Financial difficulties

The majority of caregivers interviewed voiced that they experienced financial difficulties. The most common financial challenge was the cost associated with transportation to the rehabilitation service. Six of the caregivers expressed that they frequently experienced difficulty with transportation when travelling to and from centres. The challenges included not having money to pay for transportation to the rehabilitation service:

‘… every now and then I had to borrow money … for the taxi.’ (Caregiver 9)

‘… there isn’t always money for the taxi.’ (Caregiver 12)

‘… to go there and back, we pay R100 which is too much for us.’ (Caregiver 3)

A similar situation was highlighted in South Africa more than 10 years ago by Whitelaw et al. (1993), who reported that rehabilitation at a tertiary hospital in Cape Town was a challenge owing to poor attendance caused by transport problems. Likewise, De la Cornillere (2007) reported similar findings where transport was the major problem affecting attendance at rehabilitation sessions at one of the CHCs in Cape Town. This remained a challenge even more recently when Kahonde et al. (2010) reported similar challenges.

### Caregiver and therapist relationship

Categories identified within this theme included caregiver integration in the rehabilitation process, exchange of information between the therapist and the caregiver, and trust in the relationship between caregiver and therapist. The majority of caregivers expressed that they received education and were included in the rehabilitation process:

‘… I sat in on the session …’ (Caregiver 2)

‘… they give me exercises, they write it down and draw it …’ (Caregiver 12)

‘… they were not … people that you had to be afraid of.’ (Caregiver 1)

Contrary to previous studies (Kahonde et al. 2010), the participants in this study reported a good interaction with their therapists and indicated that they received the necessary information. A review conducted by Mlenzana et al. (2013) highlighted the importance of a positive caregiver-therapist relationship.

### Facility management

Caregivers highlighted that access to rehabilitation services was not a problem even with the patients’ use of assistive devices:

‘… very easy (to access centre with wheelchair) …’ (Caregiver 1)

However, processes within the centres were a challenge and these included accessing files prior to therapy and obtaining appointments:

‘… the department where she has to go (for therapy) is there at the back. Her files are here in front. The distance is far.’ (Caregiver 3)

‘… it’s the administration that makes this a disaster.’ (Caregiver 2)

At least eight of the caregivers felt that the frequency of appointments was insufficient. Caregivers highlighted the long waiting periods between follow-up sessions:

‘… for an appointment … one month or even two months …’ (Caregiver 5)

‘They are full, now I just have to be patient …’ (Caregiver 13)

The Primary Health Care approach refers to accessibility as one of the components that is important for service delivery. Authors Brereton and Nolan (2002) confirmed in their study that, during visits to the health centres, caregivers noticed that healthcare professionals were busy and, as a result, patients had to wait longer to receive care within the health centre. Caregivers in this study were dissatisfied with the management of patients at the rehabilitation centres and expressed irritation with service delivery as things were not to their level of satisfaction during the visit. It is clear that caregivers need better service delivery from these centres as they experienced the visits as time-consuming and disorganised. This shows that the problem of overcrowding at the rehabilitation centres causes dissatisfaction regarding the service provided.

### Caregiver experience with service delivery

During the interview process, it became evident that the quality of care a caregiver is able to provide is influenced by support for his or her own physical and emotional health. The caregivers highlighted that they were not always physically capable of actively assisting the patient, and support from others was welcomed:

‘I had a friend … if I had to go somewhere, then there was someone … (to give physical assistance).’ (Caregiver 1)

In addition, it was evident that caregivers often neglected their own emotional needs. Caring for another person seems to have an impact on the caregivers’ stress levels:

‘It is a full time job. I became very sick afterwards … I did not take notice of myself …’ (Caregiver 1)

‘… sometimes it was very stressful …’ (Caregiver 9)

The findings reported by the carers regarding their physical and emotional health are similar to other studies (McLaughlin et al. 2010; Shewchuk et al. 2004) that highlighted how the impact of living and caring for people with disabilities, whether physical or mental, affects various aspects of the caregivers’ lives. Thus, although the perceptions of the caregivers regarding rehabilitation services were primarily positive, it is evident that there is a need for consideration to be given to incorporate the carer in the process of rehabilitation with a focus on ways to decrease the burden of caring. Shewchuk et al. (2004) highlighted the need for rehabilitation interventions that focus on helping caregivers develop skills and strategies to address patient-centred emotional issues.

## Limitations of the study

The findings of this study cannot be generalised for all caregivers exposed to rehabilitation services as this study was only at the primary healthcare level as it was an in-depth qualitative study. However, the findings help us to gain insight into the experience of the caregivers.

## Conclusion

The perceptions of the caregivers indicate that they do face challenges and, if we are to improve rehabilitation services, rehabilitation professionals should improve their interactions with the caregivers. Currently, there is no intervention within the rehabilitation context that addresses the needs of the caregiver. The involvement of the caregiver with the rehabilitation process within the treatment realm is evident but support for reintegration back into the society is lacking.
